# Pregnancy induces intestinal epithelial elongation and estriol-associated activation of the Hippo signaling pathway in a mouse model

**DOI:** 10.1007/s00424-025-03107-2

**Published:** 2025-08-11

**Authors:** Niklas Fritz Gängler, Cathrine Knoblauch, Franziska Hill, Bastian Lukas Zeeb, Maren Falk-Paulsen, Philip Rosenstiel, Laura Katharina Sievers

**Affiliations:** 1 Institute of Clinical Molecular Biology, Christian Albrechts University and University Hospital Schleswig-Holstein, Campus Kiel, Kiel, Germany; 2 Department of Internal Medicine IV, University Hospital Schleswig-Holstein, Campus Kiel, Kiel, Germany

**Keywords:** Pregnancy, Intestinal epithelium, Estriol, YAP, Hippo signaling

## Abstract

During pregnancy and weaning, the intestinal tract undergoes adaptations on different levels, including altered immune cell frequencies and epithelial changes. We could show in a mouse model that the overall area (crypt-villus axis length and total length) of the small intestine increased during this period of higher maternal nutrient need and that the increased area correlated with maternal weight. Quantification of cell proliferation and cell death showed an increased proliferation of epithelial cells in the lower and middle crypt. In cell culture, estriol maintained epithelial cell proliferation and progesterone-inhibited proliferation. Further, Hippo signaling is a well-known pro-proliferative pathway which integrates several upstream signals and ultimately leads to nuclear translocation of the transcription factor YAP. In the small intestine, YAP is expressed in epithelial cells, immune cells, and fibroblasts. During pregnancy and weaning, epithelial and stroma cells exhibit strong nuclear staining of YAP. Interestingly, estriol led to upregulation and increased nuclear shuttling of YAP in intestinal epithelial cell monolayers. This effect appears to be specific to the estriol treatment since the established pro-proliferative cytokine GLP-2 did not lead to increased nuclear shuttling of YAP.

**Supplementary information:**

The online version contains supplementary material available at 10.1007/s00424-025-03107-2.

## Introduction

During pregnancy and weaning in mammals, the maternal body undergoes numerous physiological changes, evolutionary developed for optimal prosperity of the offspring. Besides the genital organs, the intestinal tract is likely to be a hotspot of pregnancy-associated adaptations: (1) the need for nutrient resorption increases; (2) the maternal metabolism adapts; and (3) systemic immune cell frequencies change—all these processes are physiologically linked to the intestinal tract. However, the remodeling of the intestinal epithelium during pregnancy and weaning is still poorly understood.

The intestinal epithelium is characterized by crypts and, in the small intestine, villi, and this secondary structure enlarges the surface area, enabling increased resorptive capacity. Intestinal stem cells (ISC) at the base of the crypts are crucial for the structural integrity and functionality of the intestinal epithelium [3]. ISC regulate the constant self-renewal of the epithelium and the differentiation in specific cell types. It has been shown that nutrients and metabolic pathways regulate ISC function [13]. During pregnancy and lactation, the intestinal epithelium undergoes an elongation [1] and villous transformation [10]; however, the underlying pathways are still poorly understood.

The steroid hormones estriol and progesterone are the major pregnancy hormones. Intestinal epithelial cells express estrogen receptor (ER) [17] and ER signaling is important for the physiological architecture of the intestinal epithelium [19]. Estrogen enhances female mouse intestinal organoid regeneration via the receptor ERβ [8]. The progesterone receptor, however, is only weakly expressed in the intestinal epithelium under physiological conditions, most likely limited to mesenchymal cells [6].

The Hippo signaling pathway is an evolutionarily conserved pro-proliferative pathway integrating several different upstream signals. The pathway consists of a kinase cascade involving Large tumor suppressor kinase 1 (LATS1) and macrophage-stimulating 1 (MST1) ultimately regulating the nuclear translocation of the transcription factors Yes associated transcriptional regulator (YAP) and WW Domain Containing Transcription Regulator (TAZ). YAP and/or TAZ are essential for stem cell self‐renewal and regeneration in various tissues [13]. In the intestine, YAP is predominantly expressed in the crypts and interacts with Krüppel‐like factor 4, epidermal growth factor (EGF), and neurogenic locus notch homolog protein (NOTCH) to promote stem cell differentiation [13]. Interestingly, hormone-related expression and functional activity of YAP have been shown in endometrial epithelium [9].

The major goal of our study was to scrutinize epithelial adaptation during pregnancy and lactation. Specifically, we asked the questions: Which association of histopathological changes of small intestine and colon with weight can be tackled in a mouse model? How do the pregnancy hormones estriol and progesterone affect intestinal epithelial cell proliferation? Do we see associations of estriol effects and Hippo pathway activity?

## Methods

### Mice

Female C57BL6 mice were bred according to the guidelines of animal welfare. The animal experiments were approved prior to the study by the committee for animal welfare of the state of Schleswig–Holstein (V242-7224.121–33). After sacrifice, organs were harvested, and the length of the colon and small intestine was measured. The timepoints third trimester (T3) and after pregnancy/after weaning refer to sacrifice in the first trimester respectively 4 weeks after childbirth.

### Histological analysis

For histological analysis, the dissected mouse intestines were rolled inside out and fixed in 4% paraformaldehyde overnight at 4 °C, dehydrated, and embedded in paraffin. Two-micrometer paraffin sections were deparaffinized by xylene substitute (Thermo Fisher Scientific, Shandon) and rehydrated. Rehydrated sections were stained with H&E for morphological assessment. The lengths of the crypt-villus axis were measured using a microscope (Image Z1, Zeiss, Jena, Germany) and Zeiss Zen 3.7 software (Zeiss, Jena, Germany).

Proliferating and apoptotic cells were identified in the sections via staining of ki67 (anti-Ki-67, 556,003, BD Biosciences, Franklin Lakes, USA) and TUNEL staining (ApopTag® Plus Peroxidase In Situ Apoptosis Kit, S7101, Merck Millipore, Burlington, USA).

For quantitation of ki67- and TUNEL-positive cells, four distinct, non-overlapping fields were selected at 12, 3, 6, and 9 o’clock with three layers of intestine, cut lengthwise, each. Furthermore, the crypts were divided into equal thirds into an apical, a middle, and a basal region. Then, 10 nicely lengthwise cut crypts were selected for counting. The data was then averaged per animal. For the TUNEL staining, all positive cells in the fields of view were counted, and for the Ki-67, ten whole crypts were counted from the basal to apical compartment. The observer was blinded, without knowledge of the group of the animal, during counting the cells.

### Cell culture

Mouse Mode K intestinal epithelial cells are a crypt-derived intestinal epithelial cell line derived from the ileum of C3H/HeJ mice by introducing the SV40 large T antigen. Mode K cells grown to confluence form polarized monolayers. This polarization involves the formation of junctional complexes at the apical boundaries between neighboring cells and the development of distinct apical and basolateral membrane domains, similar to native intestinal epithelial cells. They were cultured in complete growth medium consisting of DMEM (Sigma Aldrich, St. Louis, USA) + 10% FCS (Biochrome) + 1% Penicillin–Streptomycin Solution plus, if indicated, estriol, progesterone, or GLP-2 (all from Sigma Aldrich).

### Small intestinal organoids

Intestinal epithelial cells were isolated from 6 to 12 weeks old C57BL6/J mice. To this end, the small intestine was dissected, flushed with PBS, and cut open longitudinally. The gut was then washed with PBS, cut into 3-mm pieces, and placed into a 50-ml tube containing 20 ml cold 5 mM EDTA (Invitrogen) in PBS. The tube was incubated on ice while intermittently gently shaking. Afterwards, the supernatant was discarded and replaced by 20 ml 5 mM EDTA in PBS. The incubation and replacement of the supernatant were repeated 3 times. The last time, the supernatant was replaced by PBS. The tube was then shaken vigorously for 30 s, incubated for 5 min, and again shaken for 5 min. The suspension was strained through a 100-nm cell strainer twice. The cell suspension was centrifuged at 12,000 rpm at 4 °C for 10 min. The supernatant was then discarded, and the pellet was resuspended with a solution of 240 µl PBS and 360 µl Matrigel (Sigma Aldrich, St. Louis, USA). The resuspension was plated out on a cold 24-well plate and incubated at 37 °C for 20 min. Finally, 500 µl IntestiCult medium (StemCell Tech) including supplements and 1% Penicillin–Streptomycin Solution were added to each well. The medium was changed every 2 to 3 days by discarding the old and adding new medium.

### Scratch assay

Mode K cells were seeded out on a 6-well plate for the scratch assay. To create an epithelial wound, the medium was removed and a scratch in the form of a # was made with a 100 µl/P100 pipette. Thus, four scratch crossings were formed. The cells were then stimulated with medium, estriol (0.01–1 µM), progesterone (0.1–10 µM), or estriol (0.1 µM) + progesterone (1 µM) and the wound healing progress was assessed by imaging every 24 h via microscope (Zeiss, Jena, Germany) until fully closed. Measurements were made at a virtual growth front in each corner using ImageJ (version 1.51, [12]). The average growth was analyzed.

### Viability assay

For measurement of viability and proliferation, a 3-(4,5-dimethylthiazol-2-yl)−5-(3-carboxymethoxyphenyl)−2-(4-sulfophenyl)−2H-tetrazolium (MTS) assay was used (Promega, Fitchburg, USA). To this end, cells were seeded out on 96-well plates, cultured for 24 h with medium, and stimulated for another 24 h with fresh medium, estriol (0.1 µM), progesterone (1 µM) or estriol (0.1 µM) + progesterone (1 µM). Afterwards, the MTS assay adhered to the protocol of the manufacturer. In detail, a 20:1 mixture of MTS and phenazine methosulfate (PMS) was added to the cells and metabolized by them, thus resulting in a color change. The absorbance of the samples was measured at 490 nm every 30 min with the microplate reader Infinite M200 Pro (Tecan, Männerdorf, Switzerland).

### Immunofluorescence

For the staining, cells were grown on coverslips and fixed for 10 min at 37 °C in 4% paraformaldehyde in phosphate buffered saline (PBS) after 24-h stimulation as described above. Cells were blocked at room temperature for 15 min with normal goat serum in 0.1% Triton-X-100 (Sigma Aldrich) buffered in PBS, followed by 1 h in 2% BSA in PBS. Staining was performed using YAP/TAZ antibody (1:100 in 0.1% BSA, 8418S, CST) for 1.5 h at room temperature. Next, cells were washed in PBS, followed by secondary antibody, nuclear stain, and cytoskeletal stain with Alexa Fluor 488 labeled goat anti rabbit IgG diluted 1:500 in 0.1% BSA (ThermoFischer, A11034) with DAPI (1:40,000, in PBS, D9542, Sigma Aldrich) and Rhodamine Phalloidin (1:400, T1162, Sigma Aldrich) for 45 min at room temperature. Cells were then mounted using antifade mounting media (DAKO, Hovedstaden, Denmark). Images were acquired using imager Z1 microscope (Zeiss, Jena, Germany) and analyzed with ImageJ2 Open Source processing software (Version 2.14.0/1.54f).

Quantification of fluorescence intensities was performed with Fiji open source software (version 2.14.0/1.54f): For all cells on *n* = 3 representative images, oval regions of interest in the nucleus and cytoplasm were measured, and the ratio of nuclear and cytoplasmic intensity was calculated.

### RNA-isolation, cDNA synthesis, and qPCR

Cells were directly lysed on 350 μL RLT buffer supplemented with 1% β-mercaptoethanol. RNA was isolated with RNeasy Mini Kit including DNAse digestion using the RNAse-free DNAse set (all from Qiagen, Hilden, Germany) following the manufacturer’s recommendations. RNA was quantified using NanoDrop ND-2000 and reverse-transcribed with Maxima H minus First Strand cDNA synthesis kit (ThermoFisher Scientific, Waltham, MA, USA). RT-qPCR was performed using TaqMan Gene Expression Master Mix and TaqMan® probes (Applied Biosystems, Darmstadt, Germany) in a VIIA 7 PCR system (ThermoFisher Scientific). Gene expression was obtained after relative -ΔCt quantification with ACTNB as a reference gene (housekeeping gene). Each sample was analyzed in duplicate. The primers were designed with NCBI Primer Blast as follows: (5´- > 3´):

- YAP, forward—CCCTCGTTTTGCCATGAACC;
- YAP, reverse—GTTGCTGCTGGTTGGAGTTG;
- TAZ, forward – CACGAGCTAGGCTTCGGATT;
- TAZ, reverse – TGGCTCGGGCTGAACTTCTT;
- LATS, forward – TGGTGTTAAGGGGAGAGCCA;
- LATS, reverse – TCCCAGCAACCCCAAGTATC;
- MST, forward – TTCTGTCAGCTGCATACCAGT;
- MST, reverse – ACCTAAGGGGACAATAAATAGCC.

### Data analysis

Graphs were visualized and statistical analysis was performed employing the GraphPad Prism 10 software package (GraphPad Software, San Diego, USA). The specific statistical test employed is given in the figure caption. *p*-values smaller than 0.05 were considered statistically significant; correction for multiple testing was not performed based on the small number of pre-planned comparisons.

## Results

### Pregnancy-associated weight gain correlates with an increased intestinal surface area

**Fig. 1:**
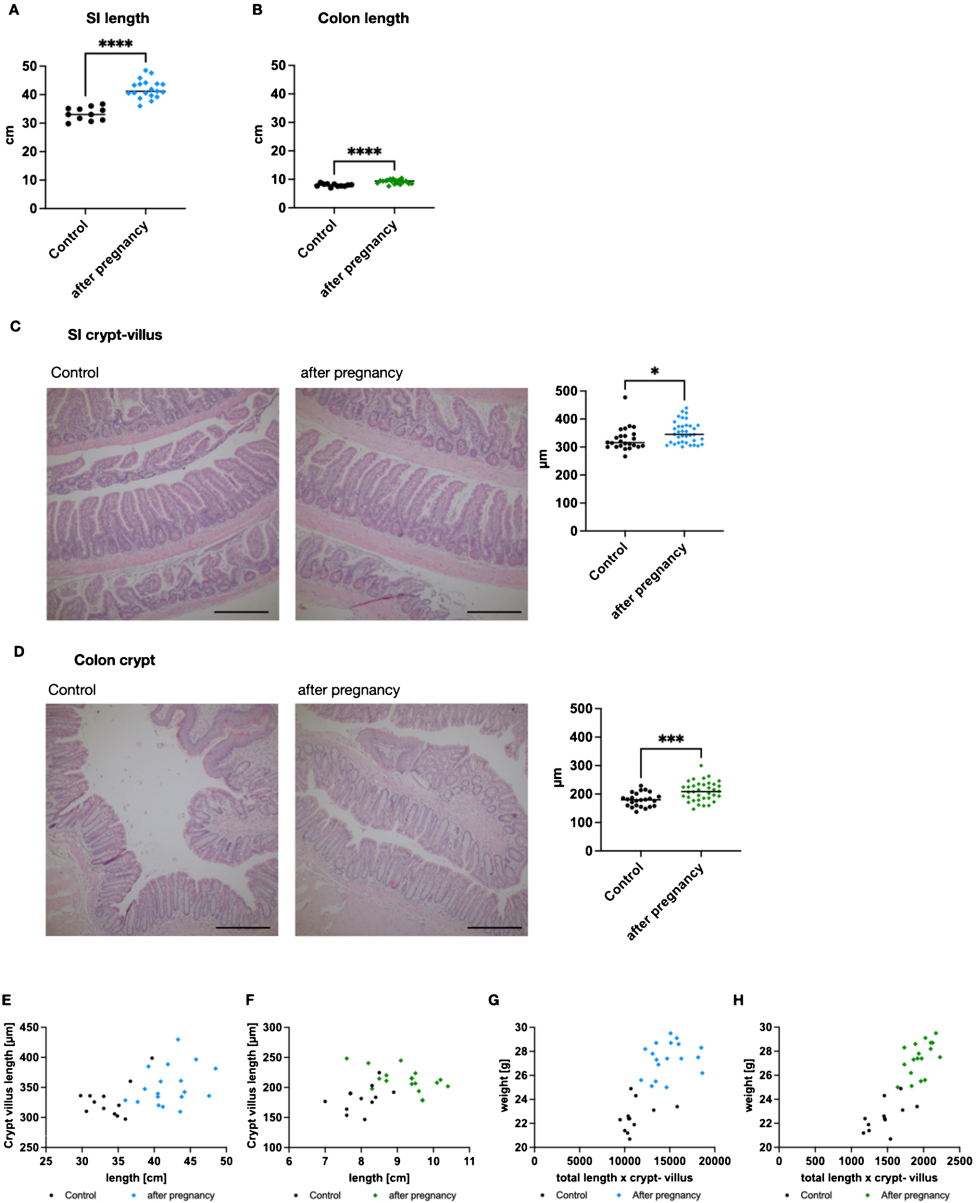
SI and colon length are increased after pregnancy. **A**, **B** The length of the small intestine (SI; **A**) and colon (**B**) was measured as the total length of the segment after sacrifice in mice four weeks after childbirth and in age-matched controls (SI and colon after childbirth *n* = 18/controI n = 12). **C**, **D** Representative images of SI (**C**) and colon (**D**), scale bar denotes 200 µm. The median SI (**C**) and colon (**D**) crypt-villus axis length was determined in histological measurements and is given as per animal mean (SI and colon after childbirth *n* = 36/contro *n* = 24). **E**, **F** For each individual animal, total SI (**E**) and colon (**F**) length and crypt-villus length are visualized (SI and colon after childbirth *n* = 18/controI *n* = 12). **G**, **H** The arithmetic product of crypt-villus length and total length was associated with the weight of the animal, visualized for SI (**G**) and colon (**H**) (SI and colon after childbirth *n* = 18/control *n* = 12). Each single dot denotes data from one animal, control *n* = 11, after pregnancy *n* = 19 for **A**–**H**. Statistical test: two-tailed *t*-test, ****p < 0.0001, ***p < 0.001, *p < 0.05

In order to examine the pregnancy-associated changes in intestinal physiology, we employed a mouse model of female mice with pregnancy and age-matched controls. In the first place, we analyzed the length of small intestine (SI) and colon and found that both SI and colon were significantly longer after pregnancy than in age-matched control mice (Fig. 1A + B). The mean SI length was 26.6% longer and the mean colon length 16.5% longer after a pregnancy than in control. Besides the total length of the segment, the length of the crypt-villus axis is a crucial determinant of resorption area. Thus, we measured the mean SI crypt-villus axis length and colon crypt length in each of the mice and found out that the difference in length was 7.2% in SI (Fig. 1C) and 16.6.% in colon (Fig. 1D). There was no clear association of crypt-villus length and total length of the segment of each individual animal (Fig. 1E and F). The capacity of nutrient resorption of the intestine relies on the resorption area and can be modified by endocrine and metabolic adaptation. In our study, the arithmetic product of total length and crypt-villus axis was calculated as an approximation of resorption area. The mean SI area was 14,684.2 × 10^−8^ m^2^ after pregnancy compared to 10,553.8 × 10^−8^ m^2^ in age-matched controls (*p* = 0.11 unpaired *t*-test, two-tailed). The mean colon area was around 13% of the SI area: 1949.1 × 10^−8^ m^2^ after pregnancy compared to 1459.9 × 10^−8^ m^2^ in age-matched controls (*p* ≤ 0.001 unpaired *t*-test, two-tailed). The approximated area of the colon correlated with the post-pregnancy weight of the animals while the correlation for SI was not statistically significant (Fig. 1G and H, slope SI pregnancy/control = 0.784; slope colon pregnancy/control = 1.522).

### Pregnancy increases proliferation in the apical crypt-villus compartment

**Fig. 2:**
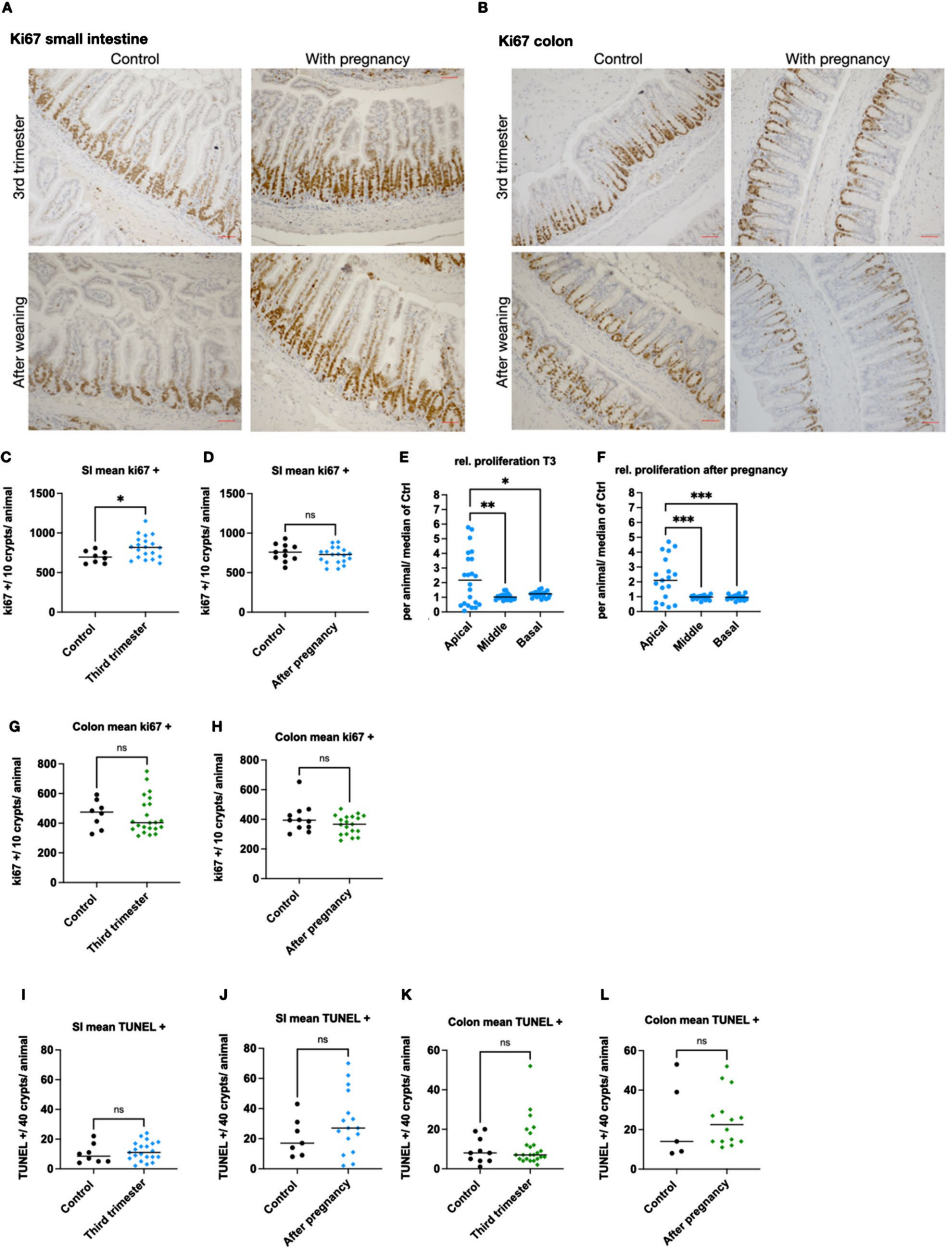
Ki67-positive proliferating cells are increased in the apical crypt. **A**, **B** Staining of SI (**A**) and colon (**B**) from mice in the third trimester of pregnancy, after weaning or age-matched controls with the proliferation marker ki67. Scale bar denotes 50 µm. **C**, **D** Count of ki67-positive cells per ten SI crypts per animal in third trimester (*n* = 22) versus age-matched control (*n* = 8) (**C**) and after weaning (*n* = 19) versus age-matched control (*n* = 11) (**D**). **E**, **F** Ratio third trimester/control (**E**) and after weaning/control (**F**) of the number of ki67-positive cells in ten SI crypts from the basal, middle, or apical compartment of the crypt. **G**, **H** Count of ki67-positive cells per ten colon crypts per animal in third trimester (*n* = 22) versus age-matched control (*n* = 8) (**G**) and after weaning (*n* = 19) versus age-matched control (*n* = 11) (**H**). **I**, **J** Count of TUNEL-positive cells per 40 SI crypts per animal in third trimester (*n* = 24) versus age-matched control (*n* = 10) (**I**) and after weaning (*n* = 16) versus age-matched control (*n* = 7) (**J**). **K**, **L** Count of TUNEL-positive cells per 40 colon crypts per animal in third trimester (*n* = 24) versus age-matched control (*n* = 10) (**K**) and after weaning (*n* = 16) versus age-matched control (*n* = 7) (**L**). Each single dot denotes data from one animal. Statistical test: two-tailed *t*-test, ***p < 0.001, **p < 0.005, *p < 0.05

Next, we wanted to find out whether the changes in crypt-villus length were associated with increased proliferation or decreased regulated cell death in the intestinal epithelium. Further, we intended to examine whether the observed elongation effect occurred during pregnancy or during weaning. To this end, histological stainings with the proliferation marker ki67 of samples from third trimester pregnancy and after weaning mice were performed (Figs. 2A and B). Quantification of ki67-positive crypt cells revealed significantly increased proliferation during pregnancy (Fig. 2C) and normalizing after pregnancy (Fig. 2D). Physiologically, most of the proliferation occurs at the base and lower parts of the crypts. During third trimester (T3), most of the proliferating cells were located at the basal and middle parts of the crypt (Fig. 2A), similar to control animals. Anyways, pregnant animals had many ki67 positive, proliferating cells in the apical compartment of the crypt (Fig. 2A). For further analyses, the crypt-villus axis was divided into three parts of equal length: a basal, a middle, and an apical third. Comparing third trimester to control, there were 2.26 times more apical, 1.04 times more middle, and 1.2 times more basal ki67-positive cells (Fig. 2E). After pregnancy, the proliferation gradually went down to 0.96 times basal, 0.95 times middle, and 2.17 times apical ki67-positive cells in SI (Fig. 2F). Apparently, during pregnancy and weaning, the proliferation zone of the SI crypt broadens and extends to upper parts, resulting in increased ki67 positivity in the upper parts. In parallel to the basal physiological proliferation zone in SI, most of the ki67-positive cells in colon samples from mice with or after pregnancy or control were located at the base of the crypt (Fig. 2B). The total number of proliferating cells was unchanged during (Fig. 2G) or after pregnancy (Fig. 2H).

Further, we quantified cell death in the histological section, which is also a physiological mechanism in intestinal epithelial hemostasis. The TUNEL staining did not show any differences between the groups (Figs. 2I–L; Supplementary Fig. 1).

### Estriol maintains intestinal epithelial cell proliferation while progesterone exerts an inhibitory effect

**Fig. 3:**
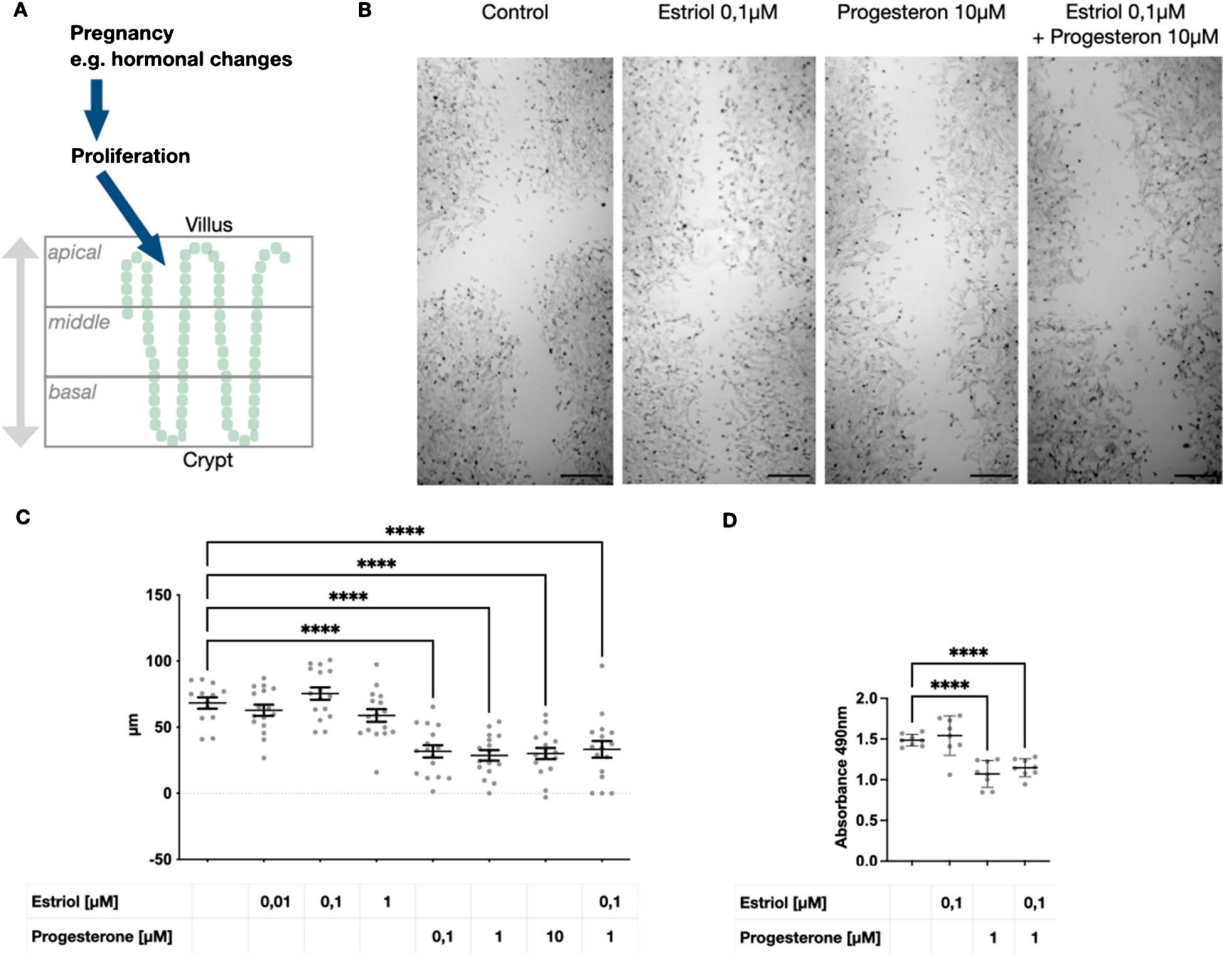
Estriol maintains the epithelial wound healing and proliferation, while progesterone has an inhibitory effect. **A** Schematic overview of the observed intestinal epithelial adaptations during pregnancy. **B** Representative light microscopy images from Mode K cell wound healing assay 24 h after scratch with 100-µl pipette tip (scale bar denotes 50 µm). **C** Quantification results from scratch assay are presented with distance of growth in micrometer on y axis (*n* = 16). **D** Further, an MTS proliferation assay where 490 nm absorbance correlates with metabolic activity was performed in Mode K cells in presence or absence of estriol and progesterone (*n* = 8). Each single dot denotes data from one culture plate. Statistical test: ANOVA, **** *p* < 0.0001

So far, we could show that pregnancy, childbirth, and weaning go along with the adaptation of the intestinal epithelium. Specifically, this effect seems to rely on increased proliferation in the apical compartment of the crypts (Fig. 3A). In vivo, pregnancy goes along with many physiological changes such as increased pregnancy hormones, altered blood circulation, regulation of the immune system, and microbiome. In order to disentangle the effects, we established an in vitro cell culture model and focused on the effect of the hormones estriol and progesterone on intestinal epithelial cell proliferation and signaling. For example, during pregnancy, estriol rises 500 times, and progesterone 10 to 20 times higher than in healthy controls. A scratch assay was performed for optical quantification of the hormonal effect on wound healing and cell proliferation. Estriol-treated cells proliferated and/or migrated well 24 h after scratch application (Fig. 3B). In comparison, progesterone-treated cells appeared to proliferate with a more dense pattern, but moved less far into the wound corridor (Fig. 3B). The total distance grown was not significantly increased in estriol-treated cells but significantly decreased by progesterone or a combination of both (Fig. 3C). The scratch assay mimics the process of cell migration during wound healing in a living organism. In contrast to the scratch assay procedure, the intestinal epithelial cell proliferation during pregnancy is not based on wound healing or epithelial cell stress. Therefore, the proliferation, respectively metabolic activity of epithelial cells during hormone exposition was determined with a proliferation assay (Fig. 3D), which confirmed the slight but non-significant increase of proliferation by estriol but inhibition by progesterone.

### Intestinal epithelial Hippo signaling is increased during pregnancy and activated by estriol

**Fig. 4:**
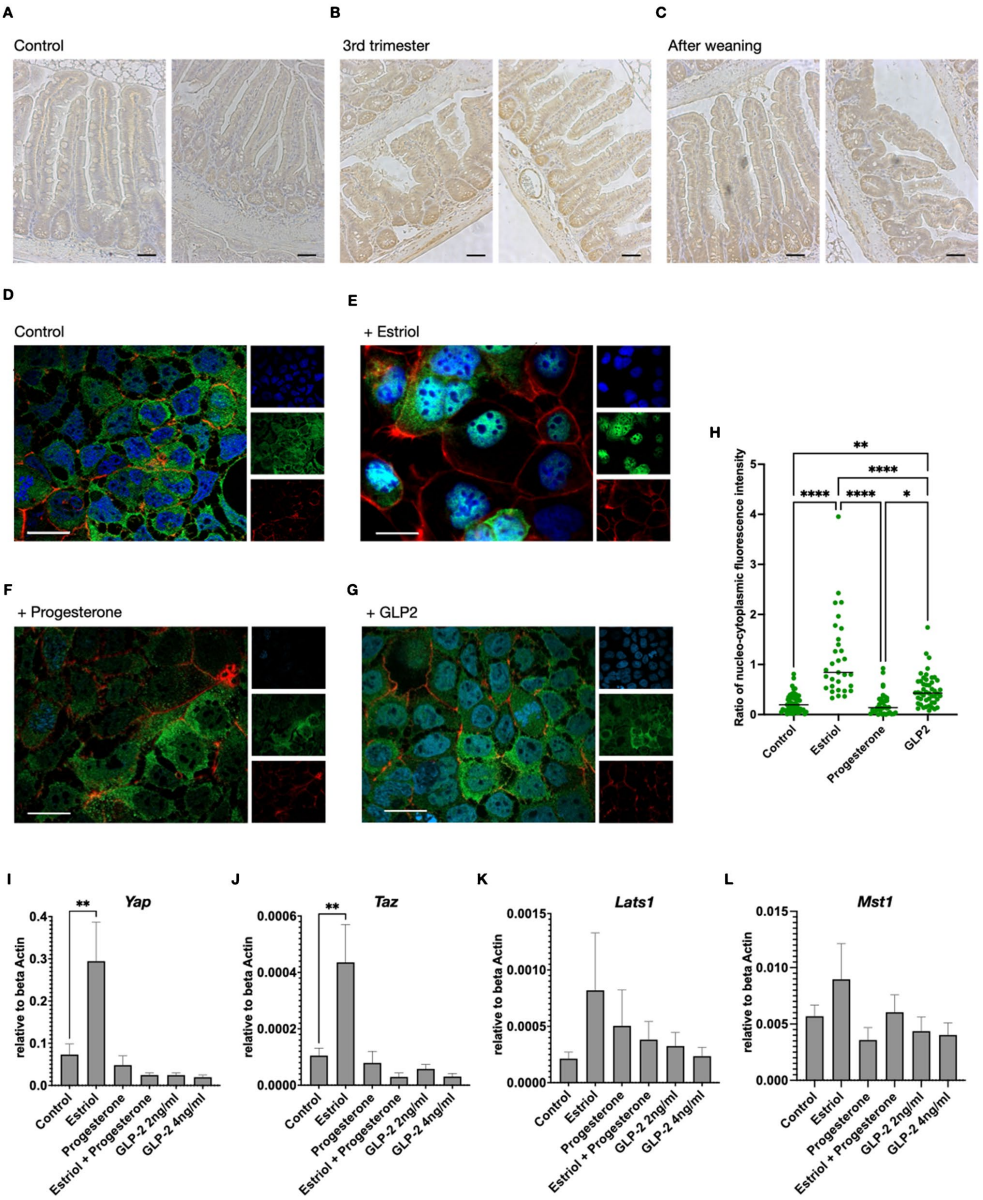
Intestinal epithelial cell hippo signaling is activated during pregnancy and by estriol. **A**–**C** Immunohistochemistry staining with anti-YAP in small intestine (SI) of control animals (**A**), during the third trimester of pregnancy (**B**) and after pregnancy and weaning (**C**). The scale bar denotes 50 µm. **D**–**G** Immunofluorescence staining of Mode K cells with anti-YAP (green), phalloidin (red), and DAPI (blue) under control conditions (**D**), after 24 h of 0.1 µM estriol treatment (**E**), after 24 h of 1 µM progesterone treatment (**F**), and after 24 h of 2 ng/ml glucagon-like peptide 2 (GLP-2) treatment (**G**). The scale bar denotes 20 µm. **H** Relative quantification of Yap fluorescence intensities in nucleus and cytoplasm from *n* = 48–55 individual cells from 3 biological replicates per condition. Statistical test: ANOVA, **p < 0.01, ***p < 0.005, ****p < 0.0001. **I**–**L** qRT PCR of *Yap* (H), *Taz* (I), *Lats1* (J), and *Mst1* (K) during indicated treatment with estriol 0.1 µM, progesterone 1 µM, or glucagon-like peptide 2 (GLP-2) 2–4 ng/ml for 24 h

Based on the results presented above, estriol might contribute to intestinal adaptation during pregnancy in vivo. It has been shown that estriol-dependent regulation of the Hippo signaling pathway contributes to breast epithelial cell fate, proliferation, and cancerogenesis. Thus, in the next step, we aimed to tackle the regulation of the Hippo signaling pathway during pregnancy-related epithelial adaptation. Scrutinizing histological staining of the Hippo pathway transcription factor Yap in intestinal epithelial samples, we observed that epithelial cells and stroma cells show a strong nuclear staining of Yap during and after pregnancy (Fig. 4A–C, for high-resolution images Supplementary Fig. 2). While the crypt cells exhibited both nuclear and cytoplasmic positivity of Yap, more apically localized epithelial cells exhibit predominantly nuclear Yap (Fig. 4B–C). In cell culture, confluent intestinal epithelial cells showed predominantly cytoplasmic staining of Yap (Fig. 4D). Treatment with estriol led to lateral expansion of the cells and visually more cytoskeletal staining at the cell margins, going along with increased nuclear Yap (Fig. 4E). Progesterone, in contrast, did not lead to increased nuclear Yap (Fig. 4F). Since glucagon-like peptide 2 (GLP-2) is a hormone with a proven pro-proliferative effect on the intestinal epithelium, we included GLP-2 stimulation in our analyses and found that increased nuclear localization of Yap is not involved in GLP-2-driven proliferation (Fig. 4G). Quantification of the nuclei-cytoplasmic ratio of fluorescence intensity supported the optical impression and showed a significant increase in estriol-treated cells (Fig. 4H). In parallel with the immunofluorescence results, estriol increased the mRNA expression of the Hippo pathway components *YAP*, *TAZ*, *LATS1*, and *MST1* (Fig. 4I–L).

**Fig. 5:**
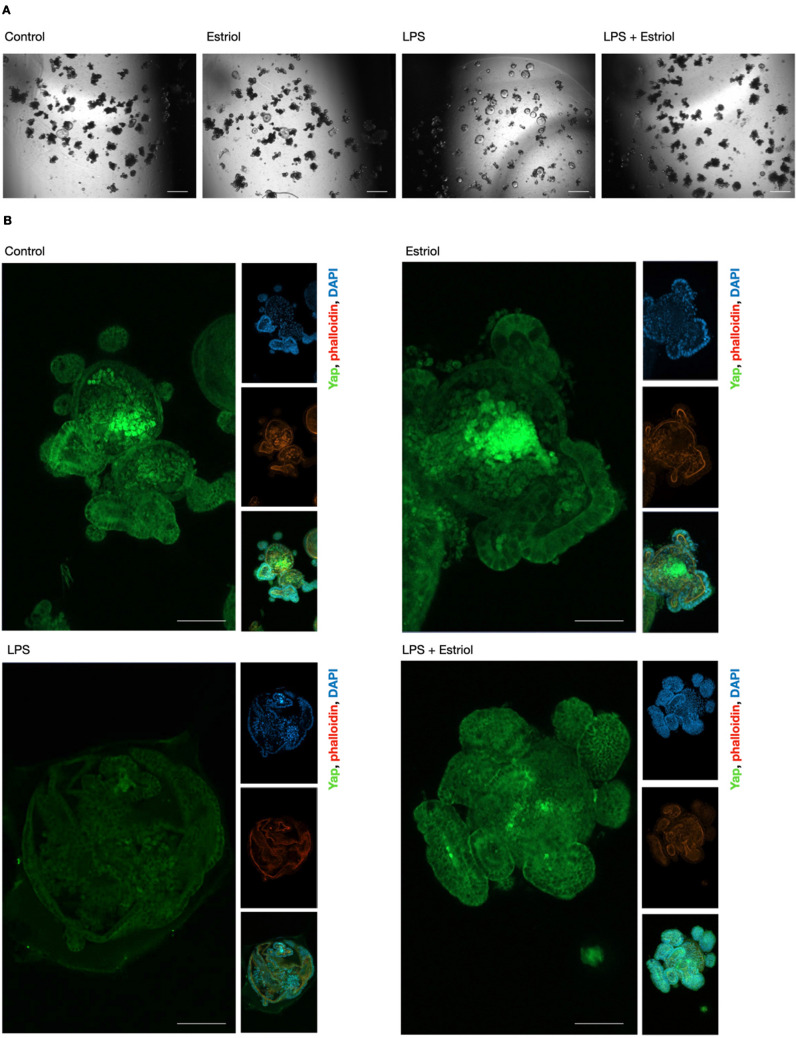
Estriol is associated with nuclear Yap staining in crypts during LPS-induced epithelial stress. **A** Light microscopy of intestinal epithelial cell organoids on day 4 after passage under control conditions and during treatment with estriol 0.1 µM and LPS 1 µg/ml. Scale bar denotes 200 µm. **B** Immunofluorescence staining of Yap (green) together with nuclear (blue, DAPI) and cytoskeletal (red, phalloidin) staining in intestinal epithelial organoids during treatment with estriol 0.1 µM and LPS 1 µg/ml. Scale bar denotes 50 µm

In a next step, we established a 3D primary culture of intestinal epithelial cell organoids, closely mimicking the in vivo situation (Fig. 5, high-resolution images Supplementary Fig. 3). On day 4 after passage, the organoids had branched and proliferated with budding (Fig. 5A). Visually, the growth was unaltered during estriol treatment. Low concentrations of LPS significantly impaired the growth and, in particular, the budding and differentiation of the organoids; however, estriol cotreatment ameliorated this effect (Fig. 5A). Immunofluorescence staining of Yap in organoids showed that, in the outer parts, Yap was located predominantly in the cytoplasm, while in the crypts of the inner part of the organoids showed a strong overall and nuclear positivity of Yap (Fig. 5B). The strong nuclear abundance of Yap in the crypts at the center paralleled the immunohistological results (Fig. 4A). Further, we evaluated whether LPS treatment, as a harmful pro-inflammatory trigger, went along with less robust Yap staining in the center of the crypts. Co-treatment with estriol normalized the morphology and the distribution of Yap in the organoid. These observations support the results obtained in cell culture experiments; however, for a more detailed understanding of sex hormone-related intestinal epithelial regeneration, further studies are needed.

## Discussion

Although intestinal epithelial remodeling might, from the evolutionary perspective of securing adequate nutrient supply for the offspring, be a hallmark of systemic adaptation during pregnancy, there have been only a few studies in mammals addressing this issue. Despite indirect evidence of remodeling such as altered metabolism and modified pro-inflammatory propensity of the intestinal epithelium, human studies on SI and colon histological architecture during pregnancy are lacking.

The finding of increased intestinal total and crypt-villus length parallels two recently and prominently published studies [1, 10]. However, our study was the first to address the physiological implications. Interestingly, both SI and colon approximated area appear positively correlated with weight in our mouse model. Besides the resorption area, gut microbiome composition and metabolic regulation might impact the nutrient resorption and weight gain. The intestinal epithelium has the capacity for nutrient sensing and mediates metabolic adaptations [15] and the gut resorptive capacity is positively regulated by increased nutrient supply [14]. Interestingly, the epithelial adaptation was not limited to the SI, where most nutrient absorption takes place, but also involved the colonic epithelium. On the one hand, this might hint at shared epithelial signaling pathways, since, e.g., vaginal, breast, and uterine epithelium also exhibit pregnancy-associated adaptations. Further, one might speculate that colonic adaptation could be influenced by microbiome changes during pregnancy migration [5, 7, 11]. In SI, we identified a broadened zone of proliferating cells in the crypt. In the colon, in contrast, proliferation or cell death were unchanged; thus, the mechanism of increased total and crypt axis length is unclear. Anyways, an elongation might not necessarily result from increased numbers of cells along the crypt but might also take place based on connective tissue softening and lateral expansion of cells with unchanged total number of cells. Unfortunately, this study was not designed to determine whether an increased nutrient intake in the first place might have caused the epithelial expansion.

Our cell culture experiments with pregnancy hormones demonstrate that estriol but not progesterone induced visual proliferation and migration of intestinal epithelial cells in a wound healing assay, of the pro-proliferative Hippo signaling pathway and increased nuclear abundance of Yap. Estriol might not alone measure up to the in vivo effect, but contribute to the physiological adaptations. Onji et al., who also observed a pregnancy and lactation-associated gut epithelial remodeling, demonstrated that this effect was at least partly explained with receptor activator of nuclear factor-κΒ (RANK) signaling in ISC [10]. Hippo signaling and RANK downstream signals can be interconnected via Ras-associated domain proteins (RASSF), which are robustly expressed in the intestinal tract [18]. Further, Hippo signaling interacts with EGF, NOTCH, and BMP signaling to balance proliferative and differentiation-inducing pathways [13].

The identification of molecular pathways during intestinal adaptation may, on the one hand, facilitate treatment of abdominal symptoms and metabolic changes during pregnancy and, on the other hand, help to develop therapeutic approaches in situations with pathologically decreased resorptive capacity, e.g. during short bowel syndrome. Based on previous studies proving that Yap/Taz are indispensable during the development of the intestinal epithelium [4], Yap induces regeneration of ISC [2] and Yap is involved in regeneration of intestinal inflammation [16] and our observations in LPS-treated organoids targeting female sex hormone-dependent regulation and the Hippo signaling pathway in intestinal epithelial regeneration might be promising.

## Supplementary Information

Below is the link to the electronic supplementary material.

ESM 1 (JPEG 1.31 MB)

ESM 2 (JPEG 1.31 MB)

ESM 3 (JPEG 697 KB)

ESM 4 (JPEG 818 KB)

## Data Availability

No datasets were generated or analysed during the current study.

## References

[1] Ameku T, Laddach A, Beckwith H et al (2025) Growth of the maternal intestine during reproduction. Cell S0092867425002004. 10.1016/j.cell.2025.02.015 10.1016/j.cell.2025.02.015 PMC12085284 40112802

[2] Cheung P, Xiol J, Dill MT et al (2020) Regenerative reprogramming of the intestinal stem cell state via hippo signaling suppresses metastatic colorectal cancer. Cell Stem Cell 27:590-604.e9. 10.1016/j.stem.2020.07.003 32730753 10.1016/j.stem.2020.07.003 PMC10114498

[3] Choi J, Augenlicht LH (2024) Intestinal stem cells: guardians of homeostasis in health and aging amid environmental challenges. Exp Mol Med 56:495–500. 10.1038/s12276-024-01179-1 38424189 10.1038/s12276-024-01179-1 PMC10985084

[4] Cotton JL, Li Q, Ma L et al (2017) YAP/TAZ and hedgehog coordinate growth and patterning in gastrointestinal mesenchyme. Dev Cell 43:35-47.e4. 10.1016/j.devcel.2017.08.019 28943241 10.1016/j.devcel.2017.08.019 PMC5823011

[5] Dang H, Feng P, Zhang S et al (2025) Maternal gut microbiota influence stem cell function in offspring. Cell Stem Cell 32(2):246-262.e8. 10.1016/j.stem.2024.10.003 39667939 10.1016/j.stem.2024.10.003

[6] Heijmans J, Muncan V, Jacobs RJ et al (2011) Intestinal tumorigenesis is not affected by progesterone signaling in rodent models. PLoS One 6:e22620. 10.1371/journal.pone.0022620 21818351 10.1371/journal.pone.0022620 PMC3144908

[7] Koren O, Goodrich JK, Cullender TC et al (2012) Host remodeling of the gut microbiome and metabolic changes during pregnancy. Cell 150(3):470–480. 10.1016/j.cell.2012.07.008 22863002 10.1016/j.cell.2012.07.008 PMC3505857

[8] Lee GS, Cody AS, Johnson KC et al (2019) Estrogen enhances female small intestine epithelial organoid regeneration. J Bio-X Res 02:9–15. 10.1097/JBR.0000000000000029

[9] Moon S, Lee O-H, Kim B et al (2022) Estrogen regulates the expression and localization of YAP in the uterus of mice. IJMS 23:9772. 10.3390/ijms23179772 36077170 10.3390/ijms23179772 PMC9456404

[10] Onji M, Sigl V, Lendl T et al (2025) RANK drives structured intestinal epithelial expansion during pregnancy. Nature 637:156–166. 10.1038/s41586-024-08284-1 39633049 10.1038/s41586-024-08284-1 PMC11666467

[11] Pronovost GN, Yu KB, Coley-O’Rourke EJL et al. (2023) The maternal microbiome promotes placental development in mice. Sci Adv 9(40):eadk1887.10.1126/sciadv.adk1887 10.1126/sciadv.adk1887 PMC10558122 37801498

[12] Schneider CA, Rasband WS, Eliceiri KW (2012) NIH image to ImageJ: 25 years of image analysis. Nat Methods 9:671–675. 10.1038/nmeth.2089 22930834 10.1038/nmeth.2089 PMC5554542

[13] Shi R, Wang B (2024) Nutrient metabolism in regulating intestinal stem cell homeostasis. Cell Prolif 57:e13602. 10.1111/cpr.13602 38386338 10.1111/cpr.13602 PMC11150145

[14] Stojanović O, Altirriba J, Rigo D et al (2021) Dietary excess regulates absorption and surface of gut epithelium through intestinal PPARα. Nat Commun 12:7031. 10.1038/s41467-021-27133-7 34857752 10.1038/s41467-021-27133-7 PMC8639731

[15] Sundaram S, Borthakur A (2021) Altered intestinal epithelial nutrient transport: an underappreciated factor in obesity modulated by diet and microbiota. Biochem J 478:975–995. 10.1042/BCJ20200902 33661278 10.1042/BCJ20200902

[16] Taniguchi K, Wu L-W, Grivennikov SI et al (2015) A gp130–Src–YAP module links inflammation to epithelial regeneration. Nature 519:57–62. 10.1038/nature14228 25731159 10.1038/nature14228 PMC4447318

[17] Thomas ML, Xu X, Norfleet AM, Watson CS (1993) The presence of functional estrogen receptors in intestinal epithelial cells. Endocrinology 132:426–430. 10.1210/endo.132.1.8419141 8419141 10.1210/endo.132.1.8419141

[18] Uhlén M, Fagerberg L, Hallström BM et al (2015) Tissue-based map of the human proteome. Science 347:1260419. 10.1126/science.1260419 25613900 10.1126/science.1260419

[19] Wada-Hiraike O, Imamov O, Hiraike H et al (2006) Role of estrogen receptor β in colonic epithelium. Proc Natl Acad Sci USA 103:2959–2964. 10.1073/pnas.0511271103 16477031 10.1073/pnas.0511271103 PMC1413854

