# Supplementary figures and images for: Pregnancy induces intestinal epithelial elongation and estriol-associated activation of the Hippo signaling pathway in a mouse model
Source: Pflugers Arch. 2025 Aug 11;477(9):1201–12. doi: 10.1007/s00424-025-03107-2 (PMC12420524; doi:10.1007/s00424-025-03107-2)

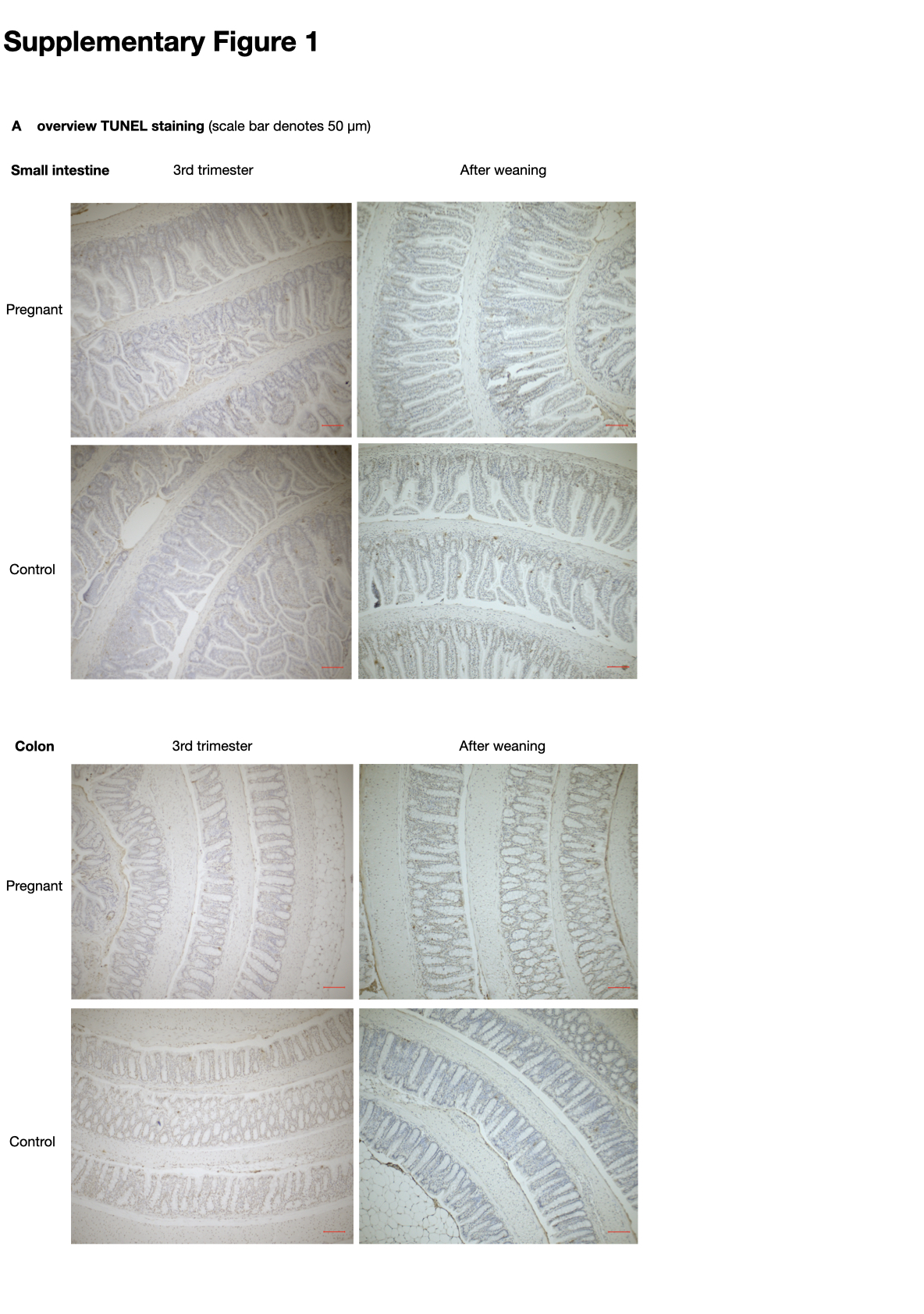

Supplement: Supplementary file 1 — (JPEG 1.31 MB) [file 424_2025_3107_MOESM1_ESM.jpeg]

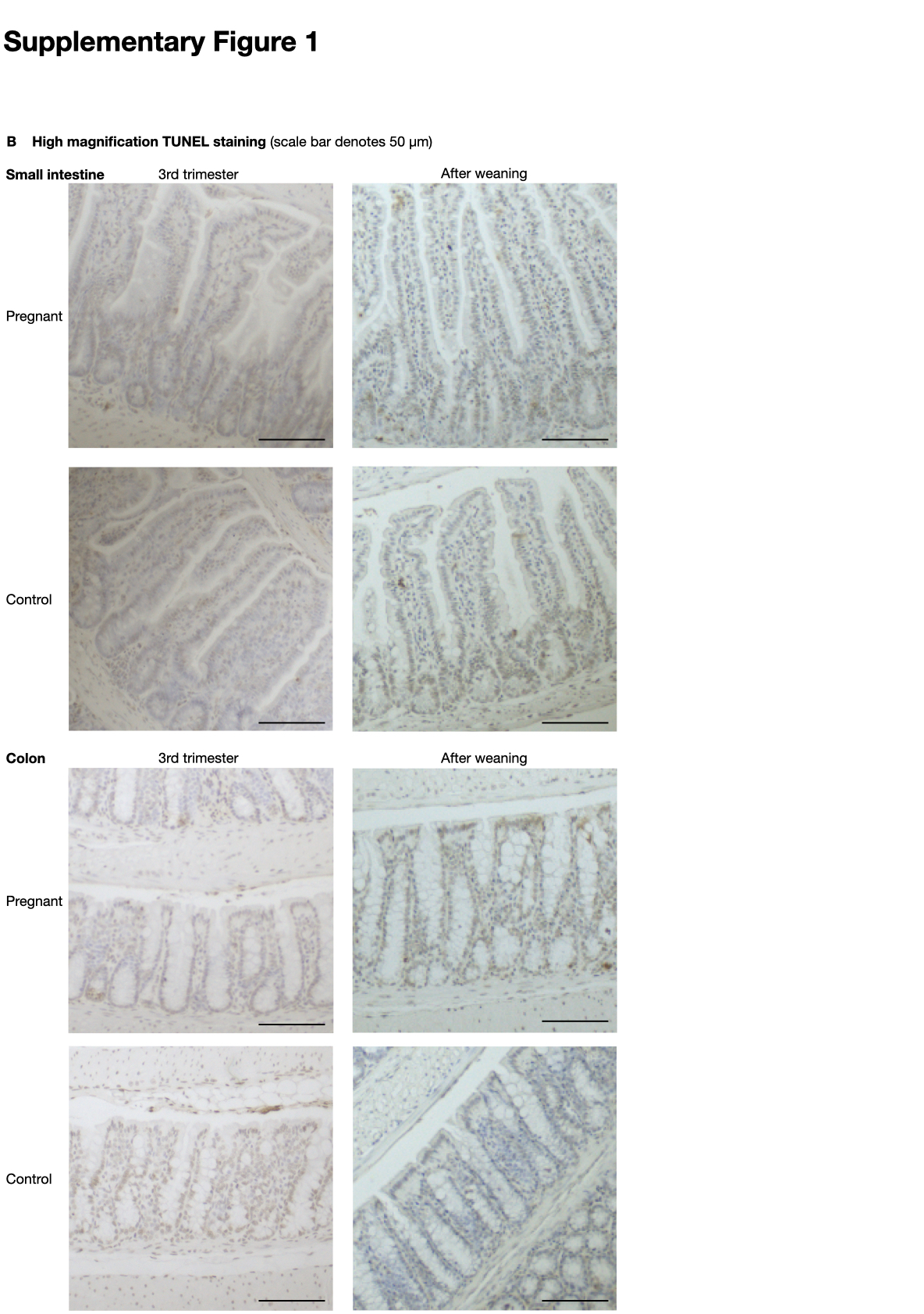

Supplement: Supplementary file 2 — (JPEG 1.31 MB) [file 424_2025_3107_MOESM2_ESM.jpeg]

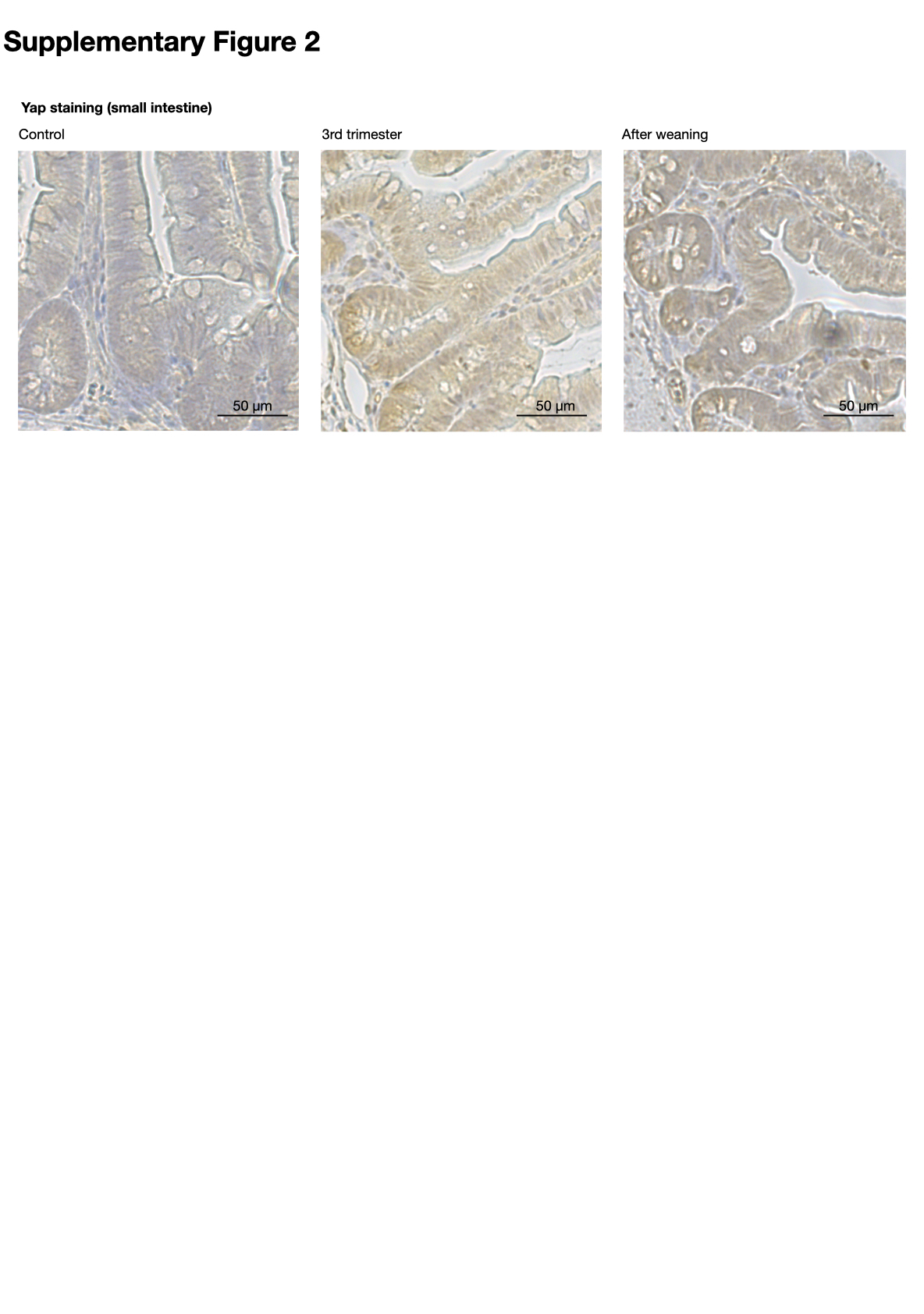

Supplement: Supplementary file 3 — (JPEG 697 KB) [file 424_2025_3107_MOESM3_ESM.jpeg]

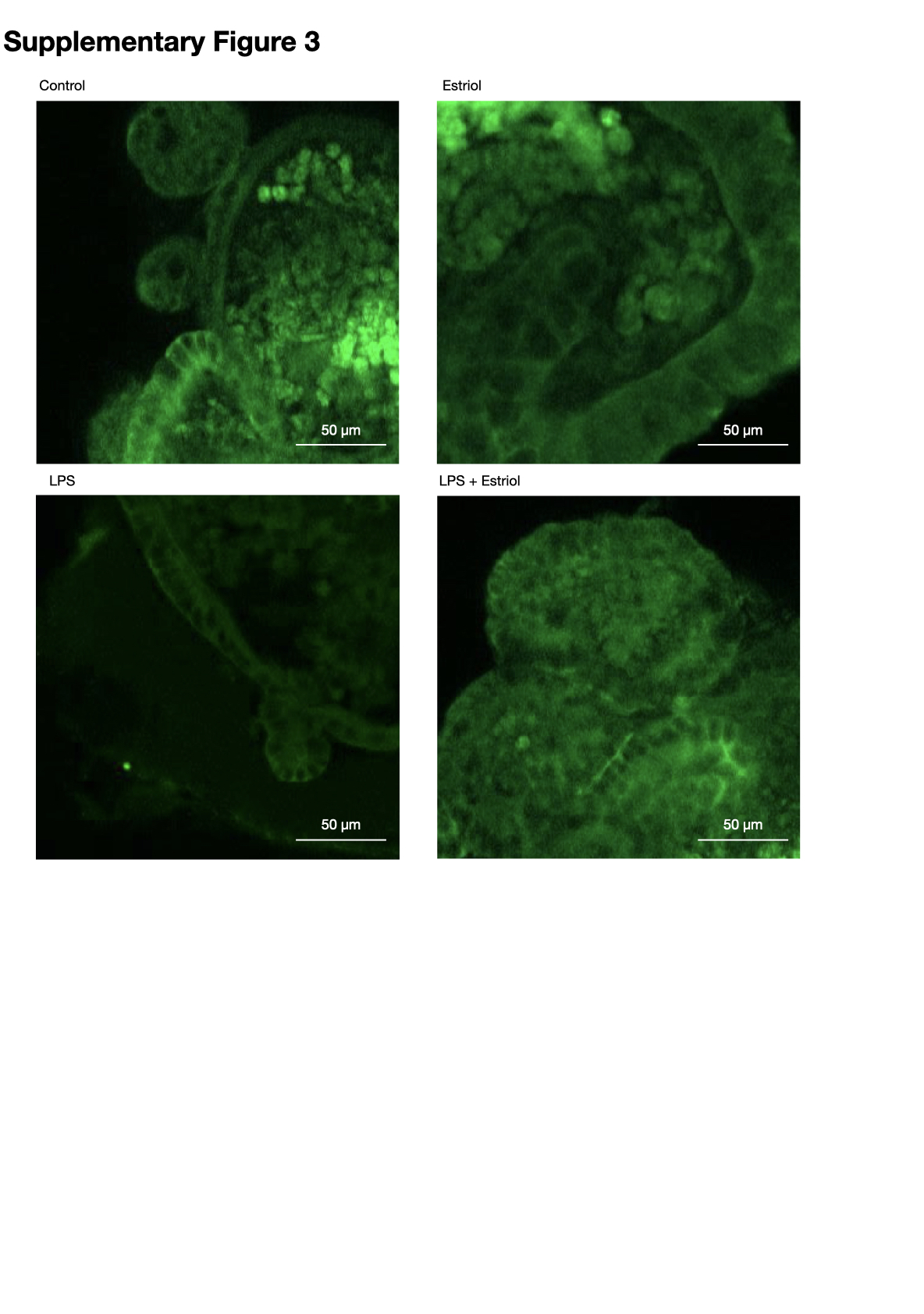

Supplement: Supplementary file 4 — (JPEG 818 KB) [file 424_2025_3107_MOESM4_ESM.jpeg]
